# Antioxidative and Anti-Inflammatory Activities of Chrysin and Naringenin in a Drug-Induced Bone Loss Model in Rats

**DOI:** 10.3390/ijms23052872

**Published:** 2022-03-06

**Authors:** Nada Oršolić, Johann Nemrava, Željko Jeleč, Marina Kukolj, Dyana Odeh, Boris Jakopović, Maja Jazvinšćak Jembrek, Tomica Bagatin, Rajko Fureš, Dinko Bagatin

**Affiliations:** 1 Division of Animal Physiology, Faculty of Science, University of Zagreb, Rooseveltov trg 6, 10000 Zagreb, Croatia; kukoljmarina@gmail.com (M.K.); dyana.odeh@biol.pmf.hr (D.O.); borisjakopovic@yahoo.com (B.J.); 2 Polyclinic Bagatin, Grada Vukovara 269a/10, 10000 Zagreb, Croatia; johann.nemrava@gmail.com (J.N.); tomica.bagatin@poliklinikabagatin.hr (T.B.); dinko.bagatin@poliklinikabagatin.hr (D.B.); 3 Department of Orthopaedic Surgery, Specialty Hospital St. Catherine, 49210 Zabok, Croatia; zjelec@yahoo.co.uk; 4 Division of Molecular Medicine, Laboratory for Protein Dynamics, Ruđer Bošković Institute, Bijenička cesta 54, 10000 Zagreb, Croatia; maja.jazvinscak.jembrek@irb.hr; 5 Department of Gynaecology and Obstetrics, General Hospital Zabok, 49210 Zabok, Croatia; rajko.fures@bolnica-zabok.hr

**Keywords:** oxidative stress, drug-induced osteoporosis, cellular antioxidative and anti-inflammatory response, chrysin, naringenin, rat model of osteoporosis

## Abstract

Oxidative stress (OS) mediators, together with the inflammatory processes, are considered as threatening factors for bone health. The aim of this study was to investigate effects of flavonoids naringenin and chrysin on OS, inflammation, and bone degradation in retinoic acid (13cRA)-induced secondary osteoporosis (OP) in rats. We analysed changes in body and uterine weight, biochemical bone parameters (bone mineral density (BMD), bone mineral content (BMC), markers of bone turnover), bone geometry parameters, bone histology, OS parameters, biochemical and haematological parameters, and levels of inflammatory cytokines. Osteoporotic rats had reduced bone Ca and P levels, BMD, BMC, and expression of markers of bone turnover, and increased values of serum enzymes alkaline phosphatase (ALP) and lactate dehydrogenase (LDH). Malondialdehyde (MDA) production in liver, kidney, and ovary was increased, while the glutathione (GSH) content and activities of antioxidant enzymes were reduced and accompanied with the enhanced release of inflammatory mediators TNF-α, IL-1β, IL-6, and RANTES chemokine (regulated on activation normal T cell expressed and secreted) in serum. Treatment with chrysin or naringenin improved bone quality, reduced bone resorption, and bone mineral deposition, although with a lower efficacy compared with alendronate. However, flavonoids exhibited more pronounced antioxidative, anti-inflammatory and phytoestrogenic activities, indicating their great potential in attenuating bone loss and prevention of OP.

## 1. Introduction

Osteoporosis (OP) is a systemic skeletal disease characterized by reduced bone mass, bone weakness, and enhanced bone fragility. There are two major categories of OP. Primary OP is the most common form, and includes postmenopausal and senile OP. It is associated with the ageing- and hormone-depletion-related gradual bone loss, and results in microarchitectural alterations of bone tissue, low bone density, and increased fracture susceptibility [1]. Secondary OP is caused by specific clinical conditions and medications. It may arise from hormonal imbalance, nutritional disorders, free radicals, cytokines, diseases (such as renal disease and cancers), or due to adverse effects of drug therapy [2,3,4,5,6,7,8]. Growing evidence suggest that various drugs, including proton pump inhibitors, glucocorticoids, selective serotonin receptor inhibitors, aromatase inhibitors, thiazolidinediones, medroxyprogesterone acetate, anticonvulsants, calcineurin inhibitors, androgen deprivation therapy, heparin, retinoic acid (13cRA or RA), and some chemotherapeutics, may increase the risk of osteoporotic fractures and induce secondary OP [2,3,4,5,6,7,8]. 

A rat model of RA-induced OP displays characteristic symptoms of human OP, and is widely used as a simple, quick, and reliable model for drug-induced secondary OP [9,10]. RA-induces osteoporotic changes and bone degradation through the increased OS levels, inflammation, hypercalcemia, increased osteoclast differentiation and function, decrease in oestrogen levels and bone mass, impaired osteoblast differentiation and reduced rate of bone formation [5,6,7,8]. RA-induced increase in OS levels promotes bone resorption by osteoclasts, resulting in decreased bone mass. RA also impairs ovarian function by lowering oestrogen levels, which is further associated with an increase in the production of pro-inflammatory cytokines in the bone marrow microenvironment [11,12]. Increased accumulation of reactive oxygen species (ROS) and accompanying inflammation are suggested as important contributing factors in OP and numerous other diseases affecting different organs [8,9]. Enhanced generation of ROS and/or exhaustion of antioxidant defence systems result in tissue damage by inducing lipid peroxidation, oxidative modifications of proteins, and oxidative DNA damage. ROS-induced oxidative DNA damage includes oxidized purines and pyrimidines, apurinic/apyrimidinic (abasic) DNA sites, double-strand breaks (DSBs), and single-strand breaks (SSBs) in different tissues, including blood, kidney, and liver, among others [13]. Moreover, ROS may induce DNA damage indirectly. In a reaction with proteins, lipids, and other cellular components, ROS may produce electrophilic species that react with DNA, further exacerbating development of diseases such as OP [14,15]. 

Considering OS as an important pathological mechanism in OP development and progression, it has been suggested that treatment with antioxidants could be effective against OP, and against OS in various tissues, including kidney, liver, spleen, and ovary [8,10,12,13].

Flavonoids comprise a heterogenous group of polyphenolic compounds. These phytochemicals possess a multiple range of health-promoting effects such as immune-stimulatory [9,13,14,15,16,17,18], oxygen radical scavenging [14,15,16,17,18,19,20], antimicrobial [21], anti-inflammatory [17,18,22], antitumor [13,18], and estrogenic activity [22,23]. 

We investigated therapeutic effects of chrysin and naringenin against bone loss in rats with RA-induced secondary OP. Flavone chrysin has been reported to possess anti-inflammatory activity by blocking expression of pro-inflammatory cytokines and antioxidant activity through the suppression of nuclear factor-κB (NF-κB) and inducible nitric oxide synthase (iNOS) in a variety of systems [8,17,19,24]. It also demonstrated potential for the treatment of osteoporosis by promoting osteogenic differentiation and mineralization in MC3T3-E1 cells through the activation of extracellular signal-regulated kinase 1/2 (ERK1/2) pathways [25]. In osteoporosis prevention rat model, treatments with chrysin or naringenin increased bone strength, bone mass, and physical bone parameters [8]. Chrysin is a compound with a flavon skeleton found in honey, propolis, royal jelly, passion flowers, and many plants [26]. Flavanones, such as naringenin and its glycoside naringin, are highly reactive compounds present in medicinal plants of families *Leguminosae*, *Rutaceae*, and *Rosaceae*, and in citrus fruits. It has been reported that naringin promotes proliferation of rat osteoblast-like cells UMR-106 and mouse osteoblastic cells, and proliferation and osteogenic differentiation of human mesenchymal stem cells. Animal studies also suggested anti-osteoporotic effects of naringin. It was shown to improve bone mineral density, increase bone mass, increase the biomechanical strength of the callus, promote bone formation but reduced resorption, and demonstrate other beneficial effects, such as antioxidant and anti-inflammatory activities [27,28]. 

The current study was conducted to examine effects of chrysin and naringenin against RA-induced secondary OP in vivo, as previous findings were mainly obtained in ovariectomy-induced OP models or in osteoblast-like cells in vitro. Effects of chrysin and naringenin on OS and markers of osteoporosis were investigated and compared with the effect of alendronate by following methods: determination of bone mineral density (BMD), bone mineral content (BMC), bone diameter, phosphorus and calcium levels in bone and serum, bone turnover markers of the femur, femoral physics characteristics and femur histological analysis, body and uterine weight, biochemical and haematological parameters, and inflammatory cytokine levels. Furthermore, we compared the efficacy of chrysin and naringenin in reducing ROS production by assessing changes in malondialdehyde (MDA) and reduced glutathione (GSH) content, and changes in superoxide dismutase (SOD) and catalase (CAT) activities in liver, kidney, spleen, and ovary, as well as severity of DNA damage by using comet assay in peripheral blood cells to obtain more comprehensive data about the potential biological activities of RA and flavonoids in different organs of osteoporotic rats. 

## 2. Results

### 2.1. Retinoic Acid Is an Effective Drug for Induction of Osteoporosis in Rats 

Verification of the success of induced osteoporosis in rats after intragastric intake of 13cRA was confirmed by the values of BMD in randomly selected rats. After 14 days, osteoporosis was successfully induced with 13cRA suspension in the rat model; the values of BMD in the proximal and distal metaphysis of the femoral neck were significantly lower in 13cRA group than in the control group (BMD proximal: 0.242 ± 0.005 vs. 0.279 ± 0.013 (*p* < 0.05); BMD distal: 0.240 ± 0.002 vs. 0.282 ± 0.012 (*p* < 0.05)).

### 2.2. Body Weight Change

**Figure 1:**
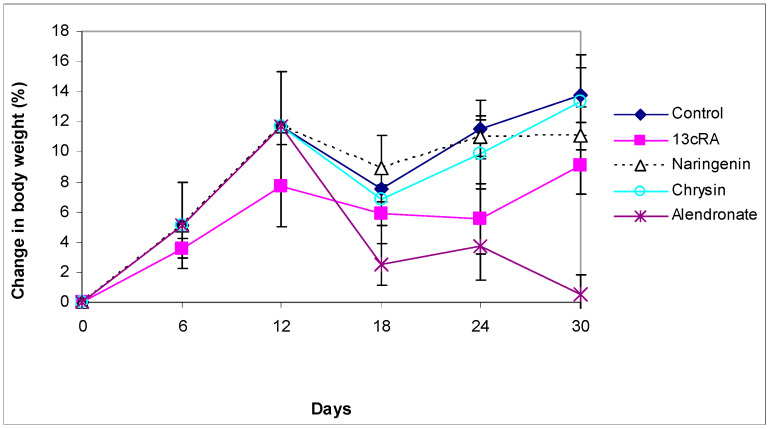
Effect of naringenin, chrysin, or alendronate on the body weight changes in rats with retinoic-acid-induced osteoporosis. Rats were administered *ig* with retinoic acid (13cRA) suspension (80 mg/kg) once daily for 14 days. After inducing osteoporosis with 13cRA, rats were administered *ig* with naringenin (100 mg/kg), chrysin (100 mg/kg), or alendronate (40 mg/kg), once daily for the next 14 days. Body weight changes (%) were calculated as follows: percentage change in weight = (final weight − initial weight) × 100/final weight. Number of rats per group: 7.

At the beginning of the experiment, the body weight (BW) was similar in all groups. The course of BW changes is shown in Figure 1. After 2 weeks of 13cRA administration, a slight weight loss was observed compared with the control group; the changes in BW in the 13cRA group on day 6 and 12 were 3.58% and 7.74%, respectively, whereas in healthy control rats the BW increased by 5.10% and 11.66%, respectively. From 18 to 24 days there was a decrease in weight in the group treated with 13cRA to 5.88 and 5.57%, respectively, while after 24 days there was a slight increase and the final change in weight in relation to the initial weight was 9.10%. Final change in body weight of control group was 13.77% in relation to initial body weight (Figure 1). After inducing osteoporosis, in rats administered with naringenin or chrysin at a dose of 100 mg/kg, a further increase in body weight was observed. From day 18 to day 30, in naringenin-treated osteoporotic rats, the body weight changed from 8.89% to 11.07%. In the same period, chrysin administered to osteoporotic rats increased body weight from 6.87% to 13.29%, respectively. No statistically significant difference was found between osteoporotic animals treated with naringenin or chrysin and rats with OP (*p* ˃ 0.05). On the contrary, at a dose of 40 mg/kg, alendronate induced a slight decrease in body weight.

### 2.3. Relative Weight Changes of Uterus, Liver, Kidney, and Spleen 

**Figure 2:**
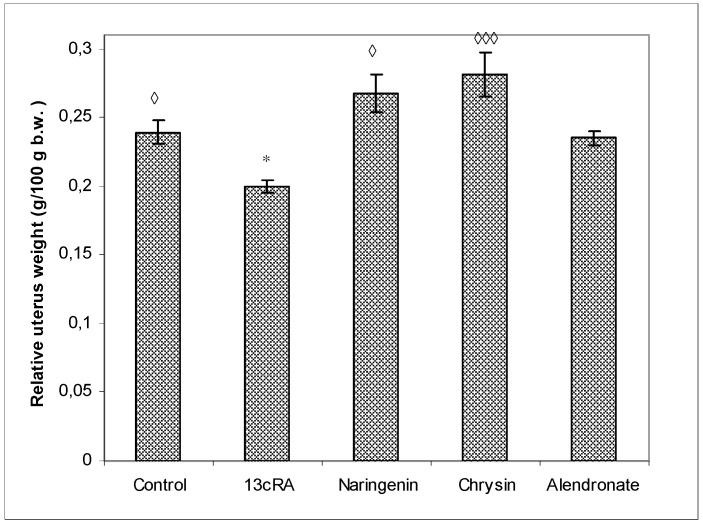
Effects of naringenin, chrysin, and alendronate on the relative uterine weight in rats with retinoic-acid-induced osteoporosis. Rats were administered *ig* with retinoic acid (13cRA) suspension (80 mg/kg) once daily for 14 days. After inducing osteoporosis with 13cRA, rats were administered *ig* with naringenin (100 mg/kg), chrysin (100 mg/kg), or alendronate (40 mg/kg), once daily for the next 14 days. Relative uterine weight was calculated as follows: relative uterine weight (g/100 g) = total uterine weight × 100/final body weight. Number of rats per group: 7. Data were analysed by the Kruskal–Wallis test. *—Statistically significant when compared with control (* *p* < 0.05). ^◊^—Statistically significant when compared with 13cRA group (^◊^ *p* < 0.05; ^◊◊◊^ *p* < 0.001).

As shown in Figure 2, the uterine weight of 13cRA-treated rats was significantly reduced when compared with osteoporotic group treated with naringenin (*p* < 0.05) or chysin (*p* < 0.001). The increase in uterine weight, as an indicator of estrogenic activity, was as follows when compared with 13cRA group: chrysin 40.71%, naringenin 25.50%, control 19.49%, and alendronate 17.43%. 

We found no statistically significant differences in relative weights of liver, kidney, or spleen (data not shown).

### 2.4. Relative Bone Weight, Length, and Diameter

**Figure 3:**
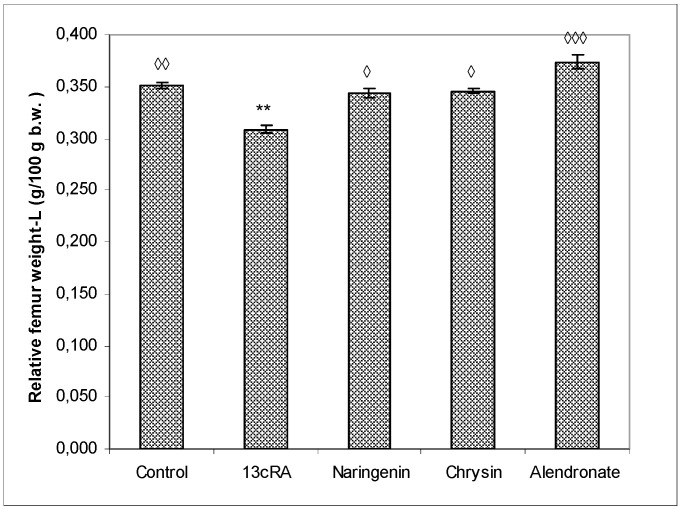
Effects of naringenin, chrysin, or alendronate on relative bone weight of the left-L femur in rats with retinoic-acid-induced osteoporosis. Rats were administered *ig* with retinoic acid (13cRA) suspension (80 mg/kg) once daily for 14 days. After inducing osteoporosis with 13cRA, rats were administered *ig* with naringenin (100 mg/kg), chrysin (100 mg/kg), or alendronate (40 mg/kg), once daily for the next 14 days. Relative bone weight was calculated as follows: relative bone weight (g/100 g b.w.) = total bone weight × 100/final body weight. Results are expressed as means of seven samples per group (*n* = 7) ± SEM. Data were analysed by the Kruskal–Wallis test. *—Statistically significant when compared with control (** *p* < 0.001). ^◊^—Statistically significant when compared with 13cRA (^◊^ *p* < 0.05; ^◊◊^ *p* < 0.001; ^◊◊◊^ *p* < 0.0001).

The relative bone weight (g/100 g b.w.) of the left femur (Figure 3) was significantly higher in healthy control group (*p* < 0.01) and the alendronate-treated group (*p* < 0.001) in comparison with the OP model group. The relative bone weight in osteoporotic groups treated with naringenin or chrysin was slightly lower than in control group (0.344 ± 0.004 and 0.346 ± 0.002 vs. 0.352 ± 0.003), but without statistical significance, indicating that naringenin and chrysin are highly effective in the prevention of 13cRA-induced bone loss. OP groups treated with naringenin or chrysin show a statistically significant increase in relative bone weight compared with the OP model (*p* < 0.05 or *p* < 0.05).

Values of the bone physical parameters of the right femur are also represented per 100 g of body weight. We measured the length of the femur (the distance between greater trochanter and medial condyle), and anteroposterior (AP) and mediolateral measurements (ML) of the proximal part, the middle part (diaphysis), and the distal part of the femur (Table 1). Statistical analysis revealed significant effect of naringenin on the femur length when compared with the 13cRA model group (*p* < 0.05). 

No significant differences were found between groups for the ML and AP diameters of the proximal epiphysis. The ML diameter in the middle part of the femur in naringenin-, chrysin-, and alendronate-treated osteoporotic rats did not differ in comparison with the control group, indicating beneficial effect test compounds in attenuating 13cRA-induced changes of the ML diameter while the ML diameter of the middle part of the bone was significantly lower in the 13cRA model group as compared with healthy control (*p* < 0.05). 

### 2.5. Ca and P Levels in the Femur and Serum

**Figure 4:**
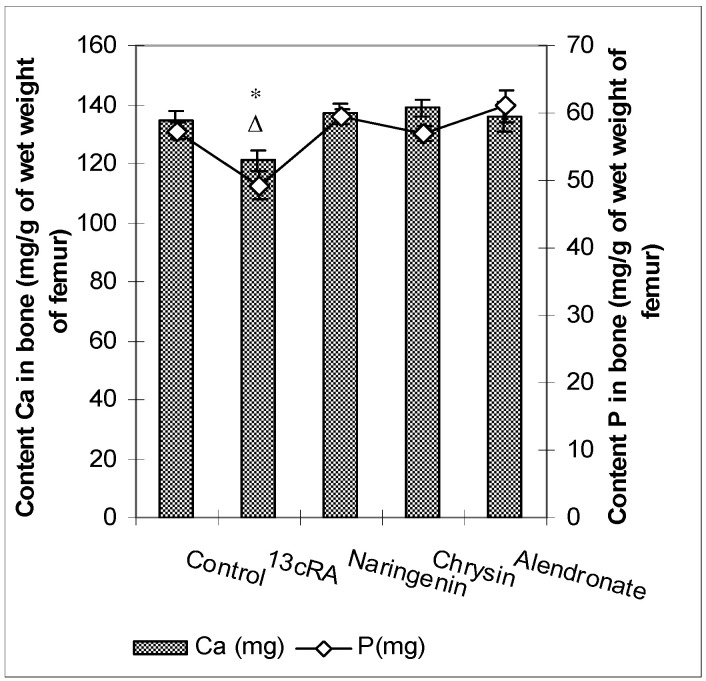
Effects of naringenin, chrysin, or alendronate on the levels of calcium (Ca) and phosphorus (P) in the rats with retinoic-acid-induced osteoporosis. Rats were administered *ig* with retinoic acid (13cRA) suspension (80 mg/kg) once daily for 14 days. After inducing osteoporosis with 13cRA, rats were administered *ig* with naringenin (100 mg/kg), chrysin (100 mg/kg), or alendronate (40 mg/kg), once daily for the next 14 days. Results are expressed as mg of calcium (Ca) and phosphorus (P) per gram of wet weight of right femurs. Results are expressed as means of seven samples per group (*n* = 7) ± SEM. Data were analysed by the Kruskal–Wallis test. *—Statistically significant difference in calcium levels compared with all experimental groups (* *p* < 0.05). ^Δ^—Statistically significant difference in phosphorus levels compared with all experimental groups (^Δ^ *p* < 0.05).

Levels of Ca and P in the femur of the osteoporotic rats (13cRA group) were significantly reduced (*p* < 0.05 and *p* < 0.05), both in comparison to healthy control and with osteoporotic animals treated with naringenin, chrysin, or alendronate (Figure 4). No statistically significant differences were found for the Ca and P content between control animals and osteoporotic groups treated with naringenin, chrysin, or alendronate. No effects of the different treatments on the serum levels of calcium and phosphorus were found (data not shown).

### 2.6. Bone Mineral Content, Bone Mineral Density, and Serum Markers of Bone Turnover

**Figure 5:**
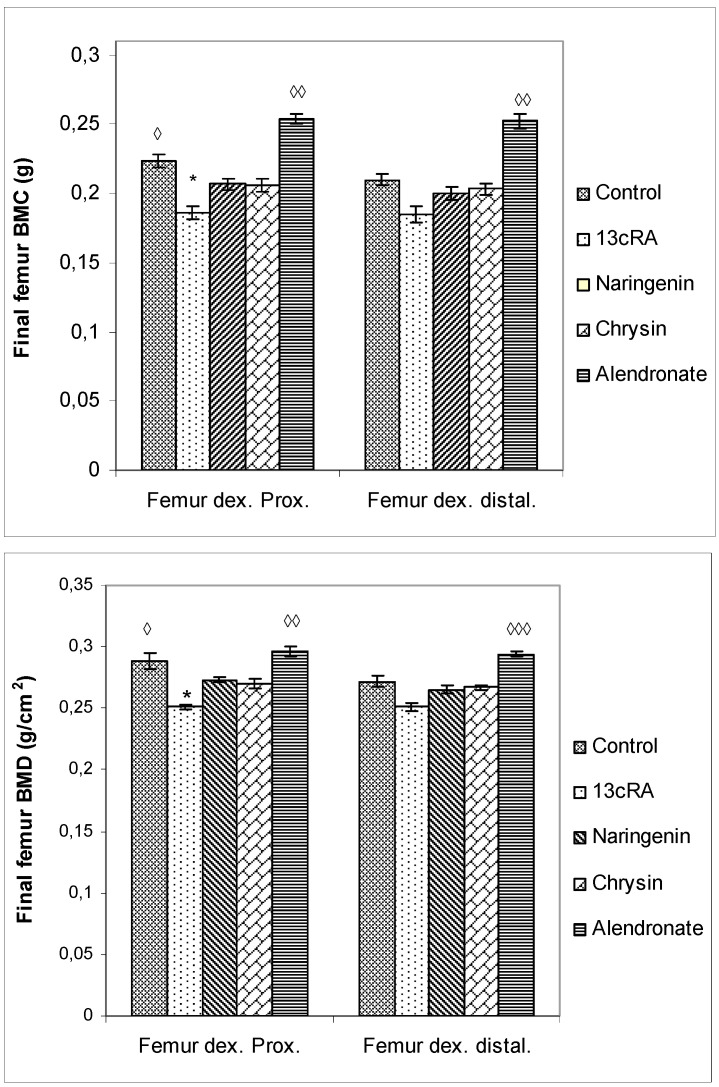
Effects of naringenin, chrysin, or alendronate on bone mineral content (BMC) and bone mineral density (BMD) in rats with retinoic-acid-induced osteoporosis. Rats were administered *ig* with retinoic acid (13cRA) suspension (80 mg/kg) once daily for 14 days. After inducing osteoporosis with 13cRA, rats were administered *ig* with naringenin (100 mg/kg), chrysin (100 mg/kg), or alendronate (40 mg/kg), once daily for the next 14 days. BMC and BMD were assessed using dual-energy X-ray absorptiometry (DXA) and left femurs. BMD was calculated as BMC divided by the measured area and reported as g/cm^2^. Results are expressed as means of seven samples per group (*n* = 7) ± SEM. Data were analysed by the Kruskal–Wallis test. *—Statistically significant compared with Control (* *p* < 0.05). ^◊^—Statistically significant compared with 13cRA (^◊^ *p* < 0.05; ^◊◊^ *p* < 0.01; ^◊◊◊^ *p* < 0.001).

BMC and BMD were measured in the proximal and distal metaphysis of the right femur. As represented in Figure 5, at day 30 BMC and BMD in proximal and distal femur metaphysis were significantly lower in the 13cRA group only in comparison with the control (Femur dex. prox. *p* < 0.05) and alendronate treatment (Femur dex. prox. *p* < 0.01; Femur dex. distal *p* < 0.01). Differences in the proximal and distal femur metaphysis part of the femur in BMD between 13cRA and alendronate-treated group was more marked than the difference in distal metaphysis part (*p* < 0.01; *p* < 0.001).

Administration of naringenin or chrysin did not improve 13cRA-induced decrease in BMC and BMD values in the proximal metaphysis region. However, a trend towards beneficial effects of naringenin and chrysin is evident for both BMC and BMD in the proximal part of the metaphysis of the femur (Figure 5).

**Figure 6:**
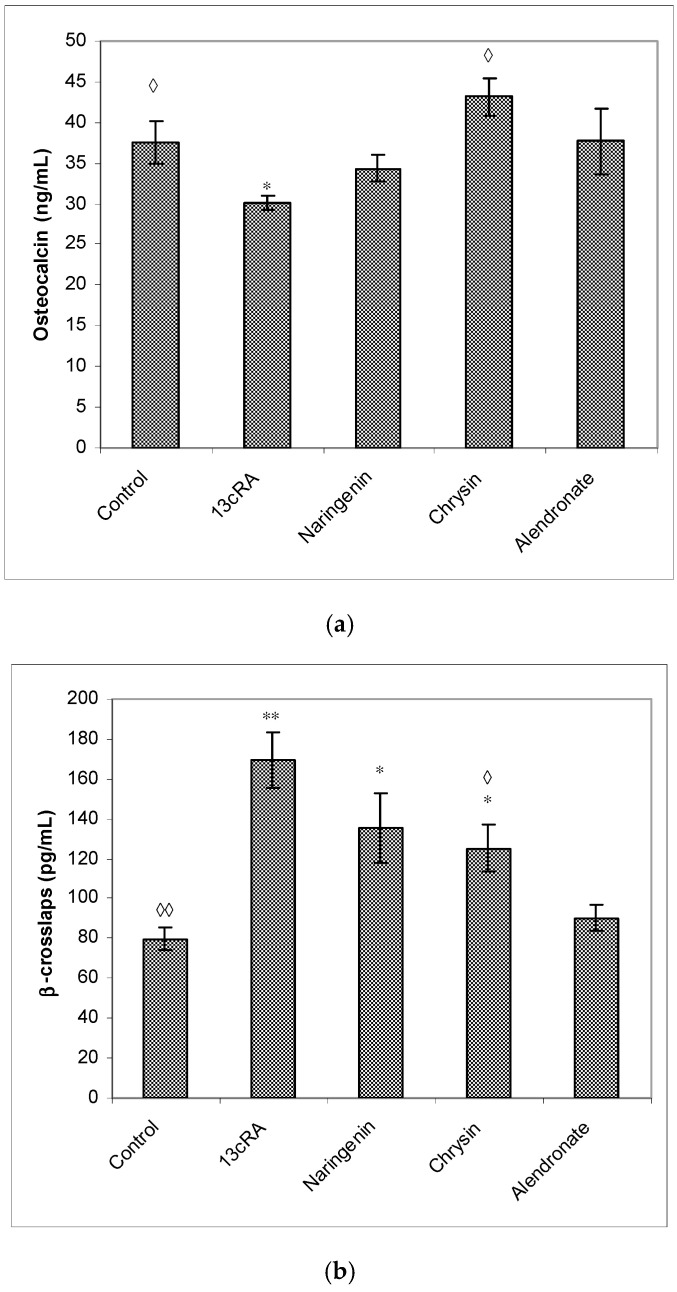
Effects of naringenin, chrysin, or alendronate on serum markers of bone turnover—osteocalcin (**a**) and β-crosslaps (**b**) in rats with retinoic-acid-induced osteoporosis. Rats were administered *ig* with retinoic acid suspension (80 mg/kg) once daily for 14 days. After inducing osteoporosis with 13cRA, rats were administered *ig* with naringenin (100 mg/kg), chrysin (100 mg/kg), or alendronate (40 mg/kg), once daily for the next 14 days. Results are expressed as means of seven samples per group (*n* = 7) ± SEM. *—Statistically significant compared with control (* *p* < 0.05; ** *p* < 0.01); ^◊^—Statistically significant compared with 13cRA (^◊^ *p* < 0.05; ^◊◊^ *p* < 0.01).

Treatment with 13cRA significantly increased serum concentration of β-CTx (*p* < 0.01) when compared with healthy animals (Figure 6b). On the contrary, OC levels were reduced in OP rats by 19.61%, and treatment with chrysin significantly (*p* < 0.05) increased OC levels and decreased β-CTx content (*p* < 0.05). Treatment with naringenin did not exert statistically significant effect on the restoration of the OC content compared with 13cRA model and healthy control (Figure 6a,b). In particular, the level of OC in the naringenin-treated osteoporotic rats was 14% higher than in the 13cRA group, but lower in the comparison with the control by 8.38% (Figure 6a). β-CTx is marker of bone degradation, and following treatment of osteoporotic rats with naringenin, chrysin, or alendronate, its concentrations were 80.14%, 74.03%, or 53.33%, respectively, of the value obtained in the 13cRA-treated animals (Figure 6b).

### 2.7. Changes in Biochemical and Haematological Parameters and Pro-Inflammatory Cytokines

Treatment with 13cRA increased ALP and LDH levels (*p* < 0.05; *p* < 0.05) (Table 2). Naringenin significantly reduced ALP, AST, and LDH values (*p* < 0.05; *p* < 0.05; *p* < 0.001) in comparison with OP model, whereas chrysin and alendronate exhibited only a trend towards lower values of these enzymes, except that in chrysin-treated animals, statistically significant decrease was observed for LDH (*p* < 0.05), whereas alendronate was capable to attenuate ALP increase (*p* < 0.01). 

No effects of the different treatments on the serum levels of metabolites, proteins, and substrates (TP—total protein; GLU—glucose; urea; creatinine; total bilirubin; CRP—C-reactive protein), except for the creatinine level in naringenin-treated osteoporotic rats in relation to healthy control (*p* < 0.05) (data not shown). 

Regarding haematological parameters, there were no significant changes observed (data not shown), except for the blood differential analyses (Table 3), which showed increased percentage of macrophages and neutrophils after alendronate treatment in comparison with healthy control group (*p* < 0.05; *p* < 0.05). 

**Figure 7:**
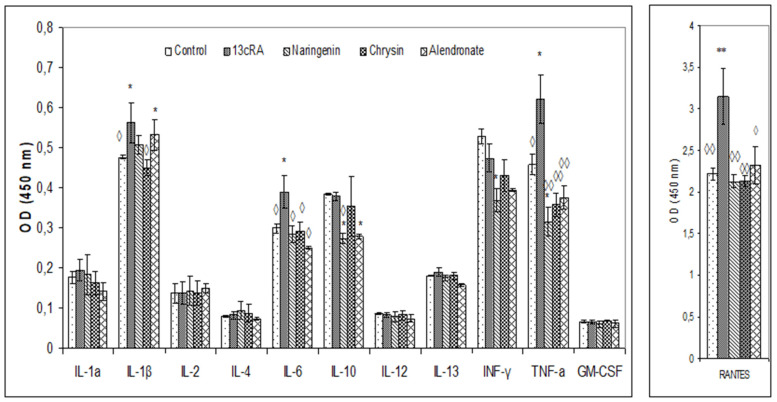
Effects of naringenin, chrysin, or alendronate on cytokine levels in rats with retinoic-acid-induced osteoporosis. Rats were administered *ig* with retinoic acid (13cRA) suspension (80 mg/kg) once daily for 14 days. After inducing osteoporosis with 13cRA, rats were administered *ig* with naringenin (100 mg/kg), chrysin (100 mg/kg), or alendronate (40 mg/kg), once daily for the next 14 days. Results are expressed as means of three samples per group (*n* = 3) ± SEM. Data were analysed by the Kruskal–Wallis test. *—Statistically significant compared with control (* *p* < 0.05; ** *p* < 0.01) ^◊^—Statistically significant compared with 13cRA (^◊^ *p* < 0.05; ^◊◊^ *p* < 0.01).

Serum cytokine analysis revealed significant changes in the 13cRA group where increased levels of IL-1β, TNF-α, IL-6, and RANTES chemokine were found, whereas naringenin and chrysin treatment induced prominent decrease in IL-1β, IL-6, TNFα, and RANTES chemokine (Figure 7). The level of RANTES chemokine was upregulated in the 13cRA group compared with the control (3.15 ± 0.38 vs. 2.21 ± 0.07), while similar values were observed in naringenin, chrysin, and alendronate groups and control animals.

### 2.8. Effect of Naringenin, Chrysin, and Alendronate on the Oxido-Reduction Status in Liver, Kidney, Spleen, and Ovary Tissues 

Table 4 shows the effects of 13cRA and naringenin, chrysin, and alendronate on OS markers in liver, kidney, spleen, and ovary cells. Retinoic acid significantly increased MDA levels in liver, kidney, and ovary (*p* < 0.05; *p* < 0.01 and *p* < 0.05) in relation to healthy control group, while the GSH content was depleted in all tissues, especially in kidney (*p* < 0.05) and ovary (*p* < 0.05). Treatment with 13cRA reduced CAT activity in all tissues but without statistical significance compared with the control group, while SOD activity was only altered in the liver compared with the control (*p* < 0.05). Naringenin reduced MDA increase in kidney and ovary of osteoporotic rats (*p* < 0.01; *p* < 0.05), and increased GSH level (*p* < 0.05) and CAT activity (*p* < 0.01) in kidney in relation to 13cRA model. Chrysin decreased MDA level in kidney (*p* < 0.01) and ovary (*p* < 0.05), while increased CAT activity in kidney (*p* < 0.01) and in ovary (*p* < 0.05). 

Treatment of osteoporotic animals with alendronate increased MDA concentrations in all tested tissues, especially in liver, kidney, and ovary (*p* < 0.01; *p* < 0.05; *p* < 0.01) in relation to healthy control, whereas GSH level was increased in kidney (*p* < 0.01) and CAT activity was increased in spleen, kidney, and ovary (*p* < 0.05; *p* < 0.001; *p* < 0.05) in relation to the 13cRA group.

### 2.9. DNA Damage of Peripheral Blood Cells

**Figure 8:**
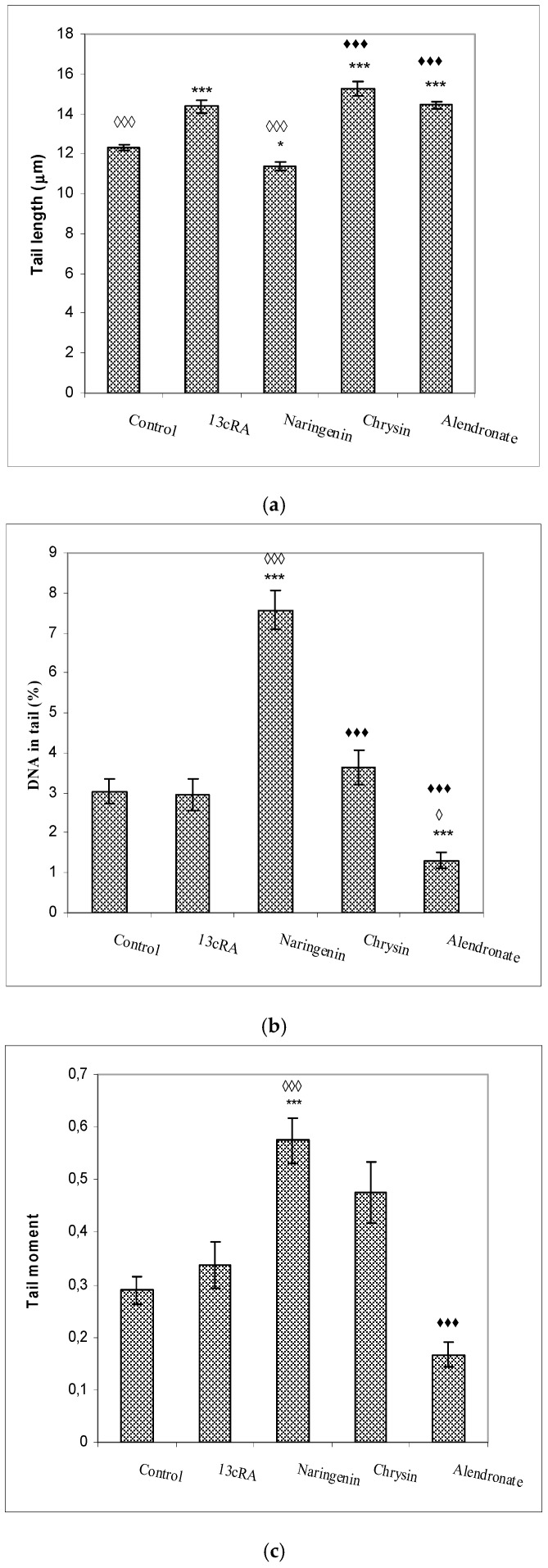
Effects of naringenin, chrysin, or alendronate on comet assay parameters (tail length (**a**), DNA in tail (**b**), and tail moment (**c**)) of peripheral blood cells in rats with retinoic-acid-induced osteoporosis. Rats were administered *ig* with retinoic acid (13cRA) suspension (80 mg/kg) once daily for 14 days. After inducing osteoporosis with 13cRA, rats were administered *ig* with naringenin (100 mg/kg), chrysin (100 mg/kg), or alendronate (40 mg/kg), once daily for the next 14 days. Number of rats per group: 7. Data were analysed by the Kruskal–Wallis test. *—Statistically significant compared with control (* *p* < 0.05; *** *p* < 0.001). ^◊^—Statistically significant compared with 13cRA (^◊^ *p* < 0.05; ^◊◊◊^ *p* < 0.001). ^♦^—Statistically significant compared with Naringenin (^♦♦♦^ *p* < 0.001).

The increased production of free reactive radicals may induce chemical modifications and damage to proteins, lipids, carbohydrates, and nucleotides. The results of the comet assay of the peripheral blood cells (tail length, tail intensity, and tail moment) are shown in Figure 8. The largest tail lengths (the distance of DNA migration from the nuclear core) were observed for the treatments with 13cRA, alendronate, and chrysin with respect to control (*p* < 0.001), while treatment with naringenin had the smallest tail length. The effect of naringenin was significant in relation to 13cRA (*p* < 0.05), chrysin (*p* < 0.001), and alendronate (*p* < 0.001), indicating that naringenin was capable to attenuate detrimental effects of 13cRA on DNA damage.

The highest values of tail intensity (% of DNA in tail) and tail moment (the product of the tail length and the percentage of total DNA in the tail) were observed following treatment with naringenin in relation to control (*p* < 0.001; *p* < 0.001), 13cRA model (*p* < 0.001; *p* < 0.001), and alendronate (*p* < 0.001), respectively, whereas alendronate reduced (*p* < 0.05) the percentage of DNA in the tail compared with 13cRA (Figure 8).

### 2.10. Histological Changes in Bone Tissue

**Figure 9:**
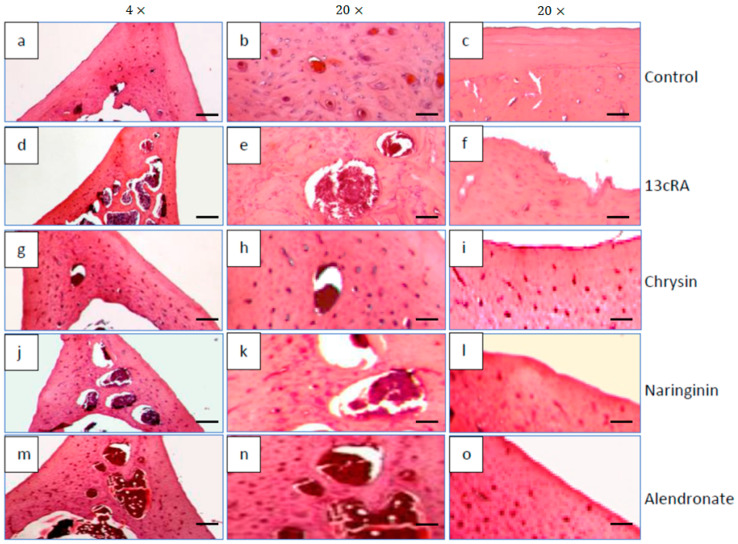
Effects of naringenin, chrysin, or alendronate on femur bone morphology in rats with retinoic-acid-induced osteoporosis. All sections were stained with H&E and examined under 4× (scalebar = 200 μm) and 20× (scalebar = 50 μm) magnification. Detailed descriptions of the images are provided in the text (**a**–**o**).

Histological examination of the distal femoral diaphysis of the control animals showed that the femoral cortex was predominantly composed of the circumferential lamellar bone tissue with lamellae and osteocytes located in round or oval cavities, while the less numbered osteons were visible in the central part of the bone tissue (Figure 9a,b), particularly at the anterior part of the cut. A few small intracortical resorption cavities filled with bone marrow, and the remaining of the “unlamellar bone tissue” were also found. Periosteum and endosteum of the bone were smooth, without any irregularities (Figure 9c).

Compared with the control group, the femoral cortex of osteoporotic rats (13cRA group) was thinned and very porous due to the formation of numerous intracortical cavities of various sizes (Figure 9d,e). Partially eroded endosteal surface of the bone was also observed (Figure 9f). The bone surface was partially lined by osteoids on the cortical side, while on the luminal surface some osteoclasts were detected. They were also found in the recesses at the eroded endosteal bone surface. Similar findings, but with slightly fewer intracortical cavities, were observed by examining the femur of osteoporotic rats treated with naringenin (Figure 9j–l).

In osteoporotic rats treated with chrysin, a marked improvement of the femoral cortical bone structure was observed (Figure 9g–i). Its thickness was almost similar to that observed in the control group although a few small intracortical cavities were still present (Figure 9h). Moreover, endosteal surface became much smoother, although small irregularities were still present. 

The cortical bone structure of the femur of osteoporotic rats treated with alendronate (40 mg/kg) was also improved. The thickness of the cortical bone was significantly higher (Figure 9m); the number and the size of the intracortical cavities were reduced (Figure 9n), while the inner surface of the cortex still contained small irregularities (Figure 9o).

## 3. Discussion

Drug-induced osteoporosis is an important and growing health issue, as many drugs have deleterious effects on bone metabolism. Drug-induced OS plays an important role in the pathogenesis of several chronic pathologies, including OP [2,3,4,5,6,7,8]. It has been shown that retinoic acid induces OP by increasing OS levels. More severe OS is associated with the reduced activities of antioxidant enzymes, such as catalase and glutathione peroxidase, in both osteoporotic patients and animals [2,3,4,5,6,7,8]. In addition, 13cRA can interact with 9-cis-RA receptors or all trans-RA receptors (RAR) in osteoblasts or act on osteoclasts to reduce expression of bone morphogenic proteins (a group of growth factors), ossein (the collagen of bones), and osteopontin (a matricellular protein). RA also stimulates osteoclast differentiation and activity, which may result in osteoporosis. So, 13cRA-induced osteoporosis is acute mode1 of OP [29,30], and mimics human OP in several important aspects, including symptoms, histomorphological features, and estrogenic response [5,6,7,8]. Oestrogen deficiency may affect antioxidant status by increasing accumulation of ROS. 

Our data demonstrated that 13cRA successfully induced osteoporotic-like changes. They were evidenced by reduced BMD of the femoral neck (Figure 5), altered geometrical and physical bone parameters (Table 1), reduced content of Ca and P (Figure 4), and changes in the levels of the bone turnover markers (Table 2 and Figure 6). Furthermore, histological examination of the femoral cortical bone demonstrated 13cRA-induced pathological changes, including the presence of various osteoporotic cavities and thinning of the bone cortex, consistent with the significant decrease in femur BMD and both calcium and phosphorous content (Figure 4 and Figure 5). Bone loss in 13cRA model could also be related to the increased number of osteoclasts and their increased activity at the luminal surface of cavities, irregularly eroded endosteal surface, and stimulatory effect of 13cRA on osteoclasts, which together results in disturbed balance between bone formation and resorption, leading to the bone loss (Figure 9). 

Oxidative damage reduces bone formation by diminishing differentiation and viability of osteoblasts, and by enhancing ROS-mediated activation of osteoclasts (Figure 3, Figure 4 and Figure 5, Table 1). Increased bone resorption by osteoclasts reduces bone mass and impairs ovarian function due to oestrogen depletion (Figure 2). The increase in osteoclasts is associated with OS, which in our study was confirmed by increased MDA levels, reduced GSH content, decreased activity of antioxidant enzymes in the liver, kidney, and ovary (Table 4), and increased levels of pro-inflammatory cytokines IL-1β, IL-6, and TNF-α, and RANTES chemokine in the serum (Figure 7). According to literature data [11,12,31], IL-1β, IL-6, and TNF-α are among the most powerful stimulants of bone resorption and are well-recognized inhibitors of bone formation. Furthermore, oestrogen deficiency also may promote increase in inflammatory mediators, including IL-1β, IL-6, and TNF-α, that further drives development of OP. Changes of IL-6 levels may reflect the severity of the inflammatory response under stress conditions, such as tissue injury, whereas IL-1β and TNF-α act synergistically to stimulate the production of other cytokines, thus promoting the imbalance of bone metabolic coupling. Increasing pro-inflammatory cytokines also contributes to the increased number of osteoclasts in bone tissue [15]. Increased production of cytokines in bone microenvironment results from the osteoclast activity, extended lifespan due to apoptosis inhibition, and regulation of osteoclast precursor cells together with their differentiation into mature osteoclasts [31]. Furthermore, released inflammatory factors (such as cytokines), in both animals and human with OP, are negatively correlated with BMD (Figure 5). RANTES is a C-C chemokine that promotes activation and recruitment of inflammatory cells such as lymphocytes, monocytes, eosinophils, and mast cells. Similar to our findings, increased expression of RANTES, a ligand for the chemokine receptors CCR1, CCR3, and CCR5 [32,33], has been observed in vivo in various inflammatory diseases, including adjuvant-induced arthritis, glomerulonephritis, granulomatous inflammation, and OP [32,33]. RANTES/CCL5 interferes with the bone metabolism leading to bone remodelling under physiological and pathological conditions through autocrine and paracrine mechanisms [32,33]. 

Excessive inflammation plays an important role in the initiation of bone, kidney, liver, and ovary damage and deterioration of their function [5,6,7,8,11]. However, these tissue changes could be modified by chrysin and naringenin treatment. Our results and literature data suggest that chrysin and naringenin may reduce tissue damage through the improved oxidative status of specific tissues, attenuated production of pro-inflammatory cytokines and increased oestrogen levels, that ultimately promotes growth and proliferation of rat osteoblasts, and increases osteocalcin levels and bone mineral deposition (Figure 2, Figure 3, Figure 4, Figure 5, Figure 6 and Figure 7). Furthermore, it seems that chrysin and naringenin may increase absorption of calcium, delay the loss of bone mass, ameliorate interactions between immune system and bone, and prevent the occurrence of osteoporosis. One of the mechanisms that may contribute to increased Ca and P absorption may be the action of flavonoids as a prebiotic on the intestinal microbiota. The mechanisms by which prebiotics promote calcium and phosphorus absorption have been described [34] as: (1) increase intestinal solubility of minerals due to bacterial production of short-chain fatty acids; (2) increase the absorption surface area by stimulating enterocyte proliferation; (3) stabilize the intestinal flora and stimulate the level of beneficial prebiotics in the intestine; (4) probiotic breakdown of phytic acid forming a mineral complex; and (5) increase expression of calcium-binding proteins. In addition, prebiotics improve bone health by: (1) releasing bone modulation factors; (2) the impact of modulating growth factors; and (3) suppression of bone resorption rates relative to bone formation rates [35], due to the antioxidant capacity of intestinal bacterial populations, such as *Bifidobacterium* and *Lactobacillus*. 

In osteoporotic rats treated with naringenin, histological analysis revealed femoral microarchitecture similar to that observed in osteoporotic control group, but with slightly fewer intracortical cavities (Figure 9). It is possible that naringenin and its metabolites act as modulators or substrates of bone extracellular matrix transport proteins, including P-glycoprotein (P-gp) and multidrug resistance protein (MRP), and that they interfere with 13cRA by reducing bone formation and regeneration processes [36,37]. On the other hand, in osteoporotic rats treated with chrysin, the cortical femoral bone structure was significantly improved (thickness similar to the control group), although a small intracortical cavities in the endosteal surface and very small erosions were still present. Besides preserving bone histology, chrysin improved changes of BMD, bone weight, length and diameters, and Ca and P content (Figure 3, Figure 4, Figure 5 and Figure 6 and Table 1). These results suggest that 13cRA-induced increase in bone resorption could be blocked by chrysin based on its inhibitory effect on osteoclast activity (Figure 2, Figure 3 and Figure 6, and Table 1), possibly due to more prominent antioxidant capacity of chrysin in relation to naringenin (Table 4) [19]. Furthermore, treatment of osteoporotic rats with chrysin significantly increased OC levels and decreased β-CTx concentrations when compared with the 13cRA model (Figure 6). OC is an important non-collagenous protein in mature bone and is highly sensitive marker of bone formation. Increased OC levels may increase osteoblast activity, thus enhancing bone formation. The underlying mechanism of these activities could be OS reduction and better preservation of estrogenic activity, as evidenced by the increased uterine weight and decreased lipid peroxidation in ovaries compared with the 13cRA model (Figure 2). As numerous studies have shown the estrogenic activity of chrysin and naringenin [25,30,38,39,40,41], we analysed only the uterine weight as a measure of estrogenic activity. The uterine weight in osteoporotic animals administered with chrysin or naringenin was higher by 40.71% and 25.5%, respectively. These results indicate the anabolic effects of chrysin by mimicking oestrogen action on osteoblasts [25], which is considered essential for the OP treatment. 

Chrysin can act as a phytoestrogen via oestrogen receptors (ER) and may induce the osteogenic differentiation of pre-osteoblast MC3T3-E1 cells by activating ERK signalling pathway. Results by Zeng et al. [25] showed that an ER antagonist ICI182780 may abrogate chrysin-induced expression of transcription factors Osterix (Runx2, Osx), Runt-related transcription factor 2, and bone formation marker proteins OCN, Col1A1, and OPN, and suppress the formation of mineralized nodules. It is known that Runx2 acts as an inducer of cell differentiation into chondrocytes or osteoblasts, whereas Osx can induce progenitor cells to osteoblastic commitment [36]. 

In addition, some studies indicated that high doses of chrysin act as a testosterone booster [37], because chrysin is a potent inhibitor of aromatase, an enzyme that converts testosterone to oestradiol. Testosterone possesses powerful anabolic and androgenic effects that are important for both men and women in maintaining bone health, particularly in elderly men. Androgens act indirectly through oestrogens, by binding to ERα and Erβ, or directly by interacting with androgen receptors (ARs). Beneficial effects of androgens on bone health are due to diminished osteoclastogenesis, enhanced osteoclast apoptosis, and prevention of osteoblast apoptosis [38]. Activation of ARs and ERα, but not ERβ, turns to be important for the acquisition and maintenance of bone mass in animal studies [38], which is consistent with our results (Figure 2 and Figure 6).

Naringenin also exerts uterotrophic effects in female mice at human-relevant doses [39], which is also evident in our data (Figure 2). By analysing uterine ERα, Breinholt and co-authors [39] found that naringenin upregulates the cytosolic concentration of ERα but decreases the nuclear concentration of ERα. The high bioavailability of citrus flavanones in animal models [40] and in humans [41], in comparison with other subgroups of flavonoids [42], suggest that flavanones (such as naringenin) may reach target cells or tissues at concentrations high enough to exert a biological activity [39]. Naringenin is one of the most consumed flavonoids in the United States and several European countries [39,40,41,42,43]. The mean daily consumption of acid fruits and juices in the United States has been estimated to be 68 g [39,43]. 

Chrysin can be isolated from honeycomb, propolis, honey, and passion flowers [44,45]. A daily intake of chrysin of 0.5 to 3 g is considered as safe [46].

Antioxidative and oestrogen-like activities of naringenin and chrysin are also involved in the increased antioxidative protection of the body. Chrysin and naringenin were capable to increase GSH levels and SOD and CAT activities in liver, kidney, spleen, and ovaries in osteoporotic rats (Table 4). This increase in antioxidative defence reduced the extent of lipid peroxidation and oxidative damage caused by the 13cRA. In addition, naringenin and chrysin suppressed the production of certain pro-inflammatory mediators. It has been shown that oestrogen prevents bone loss by blocking production of pro-inflammatory cytokines, such as IL-1, IL-6, and TNF-α, in bone marrow and bone cells [47]. On the contrary, Kacamak et al. [48] showed toxic effect of Isotretinoin (13cRA) on oocyte maturation in female rats, while Abali et al. [49] showed increased number of ovarian follicles with apoptotic granulosa cells in rats treated with Isotretinoin. It seems that Isotretinoin may be responsible for reduced ovarian reserve and toxic effects on rat ovaries, possibly through apoptosis process caused by increased oxidative stress.

Administration of chrysin or naringenin also prevented 13cRA-induced hepatotoxicity and renal toxicity, as indicated by a precipitous drop in serum AST, ALP, creatinine and LDH values (Table 2), possibly by maintaining the hepatocellular and renal cellular membrane integrity. This could be an indicator of the potential nephro- and hepato-protective efficacy of chrysin or naringenin in osteoporotic rats.

Regarding alendronate, a drug prescribed to treat OP in humans, it should be stressed that it also improved bone histology, and increased calcium and phosphorus levels and BMD after two weeks of administration (Figure 4 and Figure 5). By suppressing bone turnover, alendronate treatment prevents bone loss, preserves bone architecture, and strengthens bones. Treatment of osteoporotic rats with alendronate induced positive effect on ALP activity, but increased lipid peroxidation in kidney tissue (Table 4). Potentially, increased OS may result in kidney damage during prolonged alendronate administration. On the other hand, increased levels of antioxidant enzymes in alendronate-treated osteoporotic rats could be an indirect compensatory mechanism to protect kidneys against ROS-mediated damage. The toxic effects of bisphosphonates on kidneys and gastrointestinal system were also observed by other authors [50,51]. Accordingly, it seems that chrysin and naringenin could be a good substitute for alendronate, which exerts a strong inflammatory effect, and increases the risk of developing cancer in post-menopausal women and occurrence of atypical femoral fractures after long-term therapy [52].

Our study also confirmed that activation of immune cells, including macrophages, monocytes, and neutrophils, contributes to OS induction and free radical’s production (Table 3) in alendronate-treated rats. This increase in the number of macrophages and neutrophils leads to inflammatory response [53]. Inflammatory reactions and ROS increase mostly affected kidneys and ovaries where significant changes in OS levels were observed through MDA increase, GSH decrease, and alterations in SOD and CAT activities (Table 4). According to Benghuzzi et al. [54], alendronate and other bisphosphonates are potent osteoclast inhibitors, and their usage may lead to renal toxicity following administration of high doses, due to precipitation of bisphosphonate aggregates or complexes in the kidney. One study reported development of focal segmental glomerulosclerosis and kidney failure that were accompanied with focal mononuclear inflammatory cell interstitial infiltrate admixed with neutrophils and eosinophils [50,55,56]. It is possible that increased accumulation of free radicals ultimately leads to an increased risk of breast, uterus, and ovary cancer [47,50,51].

Besides that, ROS, such as hydroxy radical (^•^OH), can directly attack the DNA backbone and generate five classes of oxidative damage: abasic sites, oxidized bases, single-strand and double-strand breaks (SSB and DSB), formation of DNA–DNA intra-strand adducts, and DNA–protein crosslinks [13,14,15,16,17]. DNA damage is the most significant ROS-induced cellular modification, as DNA is not synthesized de novo, but it is copied, thus leading to mutations and genetic instability. Our results clearly showed that one of the underlying mechanisms of 13cRA-induced OP is OS which is closely associated with the lack of oestrogen-related osteoporotic activity [5,8,15], and that OS induces DNA damage in the peripheral blood leukocytes of 13cRA- and alendronate-treated rats (Figure 8). OS in recruited inflammatory cells exposed to alendronate increases DNA fragmentation, as indicated by the analysis of comet assay parameters (Figure 8). Increased number of neutrophils and monocytes/macrophages (Table 3) stimulates the inflammatory reactions by releasing various hydrolytic enzymes, reactive oxygen and nitrogen mediators, and many other cytotoxic agents. Increased oxygen consumption in phagocytes via “oxidative, respiratory or metabolic burst” can be useful for killing bacteria and tumour cells, but increased presence of ROS can also damage a healthy tissue. Such a negative microenvironment may result in kidney injury, gastrointestinal adverse effects, cancer, jaw necrosis, and other inflammatory reactions [47,50,51,52,53]. Naringenin and chrysin as antioxidative and anti-inflammatory agents could exert beneficial effects and offer protection against OS-induced diseases such as OP.

## 4. Material and Methods

### 4.1. Reagents

Intragastric application (*ig*) of isotretinoin (13cRA; 13 cis-Retinoic acid) (Roaccutane^®^, Hoffmann-La Roche Ltd., Basel, Switzerland) was used for the induction of OP in Y59 female rats. 13cRA was administered at a dose of 80 mg/kg for 14 consecutive days. Alendronate (Alendor^®^70, Pliva, Zagreb, Croatia), a drug used to treat OP, was used as a positive control. It was also applied *ig* during 14 consecutive days, but at a dose of 40 mg/kg [8,15]. Narketan^®^ (100 mg/mL, provided by Vetoquinol S.A. BP 189, Lure Cedetx, France) and Xylapan^®^ (20 mg/mL, provided by Vetoquinol Biowet Sp., Gorzow, Poland) were used to induce anaesthesia.

### 4.2. Chrysin and Naringenin

Chrysin (5,7-dihyrdoxyflavon; ≥96.5%) and naringenin (5,7-Dihydroxy-2-(4-hydroxyphenyl)chroman-4-one; ≥95%) were purchased from Sigma-Aldrich Ch. Co. Inc. Milwaukee, WI, USA. Before application, chrysin or naringenin were dissolved in ethanol and further diluted in water. The final concentration of ethanol was 0.5% (*v*/*v*). Ethanol (0.5%) was used in the control group. Chrysin or naringenin were given *ig* during 14 consecutive days at a dose of 100 mg/kg body weight [8].

### 4.3. Experimental Animals, Study Design, and Organ Processing 

A total of 50 Y59 female rats, three months old, were obtained from the Department of Animal Physiology at the Faculty of Science, University of Zagreb. The ethical committee (Faculty of Science, University of Zagreb, Croatia) approved the study (Approval Code: 251-58-10617-14-28, Date of Approval: 10 June 2014). All the procedures were carried out in accordance with the approved ethical guidelines, including the law of the Republic of Croatia (Law on the Welfare of Animals, NN135/06 and NN37/13), EU Directive 2010/63/EU for animal experiments (reference: OJEU 2010), and the Guide for the Care and Use of Laboratory Animals, DHHS Publ. # (NIH) 86-123, and were in compliance with the ARRIVE guidelines.

Following acclimatization, rats were divided into 5 groups: control group, 13cRA-treated group, and 13cRA + chrysin, 13cRA + naringenin, or 13cRA + alendronate group. At first, OP was induced in 40 animals by *ig* administration of 13cRA at a dose of 80 mg/kg, once daily for 14 consecutive days. After induction of OP, rats were divided into 4 groups which were treated *ig* for another 14 days: 1st group—osteoporotic model treated with 0.5% ethanol in physiological solution; 2nd group—osteoporotic model treated with chrysin at a dose of 100 mg/kg [15]; 3rd group—osteoporotic model treated with naringenin at a dose of 100 mg/kg [15]; and 4th group—osteoporotic model treated with alendronate at a dose of 40 mg/kg [15]. The 5th group were control healthy animals that were treated *ig* with physiological solution. Development of OP was confirmed by measuring bone mineral density (BMD) in comparison with the control group.

All rats were fed a standard laboratory diet (4 RF 21, Mucedola, Settimo Milanese, Italy) and were housed under standard conditions (12/12 h light–dark cycle, room temperature around 25 °C and 60% humidity), with free access to food and water. Body weights of the animals were recorded daily, and the dosing was adjusted accordingly. At 4 weeks after the treatment, rats were anesthetized using a mixture of ketamine at a dose of 75 mg/kg with xylazine at a dose of 10 mg/kg. For haematological and biochemical analysis, blood samples were taken from the axillary blood vessels of anesthetized rats. The blood was placed on ice and the serum was separated by centrifugation. Both femurs were then collected, carefully cleaned from the soft tissue, weighed, and their lengthwise dimensions were measured with callipers. The right femurs were used for the analysis of bone calcium (Ca) and phosphorus (P) levels. The left femurs were used for histological analysis and the determination of the bone mineral density (BMD). The uterus of each rat was dissected and weighed. Liver, kidney, spleen, and ovary tissues were collected to determine oxidative and antioxidative status by measuring extent of lipid peroxidation, GSH levels, and SOD and CAT activities. All parameters measured and methods used are briefly summarized in Table 5.

### 4.4. Monitoring Weight Changes of Animals and Selected Organs

The animals were weighed on digital scale at 6 days intervals (at experimental day 0, 6, 12, 18, 24, and 30). The weight change of animals and the relative organ weights (uterine, liver, kidney, spleen, and femur expressed in g/100 g) were calculated by following formulas:

$$\mathrm{Percentage}\;\mathrm{change}\;\mathrm{in}\;\mathrm{weight}=\frac{\left(\mathrm{Final}\;\mathrm{weight}-\mathrm{Initial}\;\mathrm{weight}\right)\times 100}{\mathrm{Final}\;\mathrm{weight}}$$

$$\mathrm{Relative}\;\mathrm{organ}\;\mathrm{weight}\;\left(\frac{g}{100\;g}\right)=\frac{\mathrm{Total}\;\mathrm{organ}\;\mathrm{weight}\;\times 100}{\mathrm{Final}\;\mathrm{weight}}$$

### 4.5. Bone Harvesting and Analysis of Bone Physical Parameters 

After sacrificing the animals, the right femurs of the rat were dissected, cleaned, and immersed in saline solution to prevent dehydration for the subsequent measurement of bone wet weight, length, bone diameters (the distal and proximal epiphyseal diameters of the femur were measured in mediolateral (ML), and anteroposterior directions (AP) by digital calliper), BMD, and analysis of Ca and P levels. Bone harvesting and analysis were performed as previously described [15]. Ca was determined by atomic absorption spectrophotometry Varian Spectra AA-300, (Varian Inc., Palo Alto, CA, USA) at a wavelength of 422.7 nm, whereas P was determined spectrophotometrically at 660 nm (Cary 50; Varian Inc., Palo Alto, CA, USA). Results are expressed as mg Ca or P/g wet tissue weight. BMD was determined on Hologic QDR^®^ 4000 DXA machine (Hologic Inc., Zaventem, Belgium) by dual-energy X-ray absorptiometry (DXA). Briefly, the area of proximal and distal parts of the left neck of femur (thigh bone metaphysis) were subjected to measurement to obtain the values of bone area (cm^2^, area) and bone mineral content (BMC, g). Data on bone mineral density (BMD) were calculated from the relationship of these parameters (g/cm^2^). The same technician performed all BMD measurements. During the analysis of these parameters, the manufacturer’s instructions were followed in order to assess long-term reproducibility of the measured values. The coefficient of variation (QC) for BMD and femoral bone amounted to 1.15% or 1.1% (0.61%).

### 4.6. Serum Markers of Bone Turnover

The serum levels of bone turnover markers, osteocalcin (OC) and β-CrossLaps (β-CTx), were determined by using the N-MID Osteocalcin kit (Cobas-Roche, Switzerland) and β-CrossLaps kit (Cobas-Roche, Switzerland), respectively. OC and β-CTx were analysed with the electrochemiluminescence immunoassay “ECLIA” and immunoassay analyser Elecsys 2010 (Hitachi High-Technologies Corporation, Tokyo, Japan) according to the manufacturers’ instructions.

### 4.7. Analysis of Haematological and Biochemical Parameters

The whole blood samples were collected into vacutainer tubes without anticoagulant and after separation of serum by centrifugation, serum aspartate aminotransferase (AST), alanine aminotransferase (ALT), alkaline phosphatase (ALP), glutamyl transferase (GGT) and amylase, urea, creatinine, blood glucose, lactate dehydrogenase (LDH), total protein, serum calcium (Ca), and phosphate (P) levels were analysed with the Beckman Coulter AU680 (USA) clinical chemistry analyser.

The whole blood was collected into the plastic tubes with K2EDTA as an anticoagulant. Several routine haematological parameters were quantified: number of erythrocytes (E), mean corpuscular volume of red blood cells (MCV), haemoglobin (Hgb), haematocrit (Hct), mean corpuscular haemoglobin (MCH), mean corpuscular haemoglobin concentration (MCHC), number of leukocytes (L), and platelet count (Plt). Blood specimens were analysed using standard laboratory methods and blood cell counter Cell-Dyn ^®^ 3700 (Abbott, Chicago, IL, USA). 

### 4.8. Histological Analysis

A detailed histomorphological analysis of the left femur was evaluated following standard haematoxylin and eosin (HE) staining method [15], and sections taken from comparable areas were examined by optical microscopy (Nikon Eclipse E600, Melville, NY, USA) at 40, 100, and 200× magnifications. 

### 4.9. Tissue Preparations for Quantitative Analysis of Oxidative Stress Biomarkers

Liver, kidney, spleen, and ovary samples were prepared as previously described [15]. Briefly, tissue homogenates were centrifuged by Micro 200R centrifuge (Hettich, Westphalia, Germany) for 15 min at 10,000 rpm and +4 °C, and the obtained supernatants were used for the assessment of GSH and malondialdehyde (MDA) content, and SOD and catalase CAT activities, by routinely used and well-established methods [8,15,17,18]. The values of all investigated parameters were normalized to the protein concentration of each sample.

Briefly, the extent of lipid peroxidation was estimated by determining the concentration of MDA. MDA in a reaction with thiobarbituric acid produces a pink chromogen that absorbs at 532–535 nm. A 200 μL of supernatant was mixed with 200 μL of 8.1% aqueous sodium dodecyl sulphate (SDS), 1.5 mL of 20% acetic acid (pH 3.5), and 1.5 mL of 0.81% aqueous thiobarbituric acid. The mixture was incubated for 60 min at 95 °C. After cooling the samples on ice, the absorbance was recorded at 532 nm with Libra S22 spectrophotometer (Biochrom, Cambridge, UK) [15]. The MDA content was expressed as mg MDA/mL/mg protein.

To estimate GSH content, 200 μL of 3 mM 5-5′-dithiobis [2-nitrobenzoic acid] (DTNB, Ellman’s Reagent, Sigma-Aldrich Ch. Co. Inc. Milwaukee, WI, USA) was added to 30 μL of sample supernatant. In a reaction of DTNB with GSH, a yellow chromophore 5′-thio-2-nitrobenzoic acid (TNB) with absorbance maxima at 412 nm, and mixed disulphide GS-TNB are formed [8,15]. GS-TNB is further reduced by glutathione reductase to GSH and more TNB, thus enhancing the sensitivity of the assay. Various concentrations of GSH were used to obtain standard curve for the GSH quantification. 

SOD activity was estimated by measuring inhibition of the cytochrome c reduction by superoxide anion which is generated during xanthine oxidation by xanthine oxidase (optimized reaction ratio ΔA/min ≈ 0.025). SOD competes with the superoxide anion and inhibits cytochrome c reduction. The appearance of reduced cytochrome c is measured spectrophotometrically at 550 nm. The percentage of inhibition was calculated using a calibration curve based on standard SOD enzyme dilutions [15]. For the determination of SOD activity, 25 μL of undiluted sample was added to 1.45 mL of the reaction mixture containing 0.05 mM cytochrome c and 1 mM xanthine. A measure of 20 μL of xanthine oxidase (0.4 U/mL) was added to start the reaction which was monitored for 3 min at 550 nm [15]. 

CAT activity was estimated by monitoring the rate of H_2_O_2_ disappearance at 240 nm. The reaction mixture (total volume of 1 mL) contained 980 µL 10 mM H_2_O_2_ in phosphate buffer (pH 7.0) and 20 µL PBS or sample. Catalase activity was calculated based on the extinction coefficient of H_2_O_2_ (ε = 39.4 m/M cm); the specific activity was expressed as U/mg protein.

### 4.10. Analysis of Inflammatory Cytokines 

The analysis of 12 pro-inflammatory cytokines and chemokines (IL1α, IL1β, IL2, IL4, IL6, IL10, IL12, IL17A, IFNγ, TNFα, GM-CSF, and RANTES) was performed in serum samples using Multi-Analyte ELISArray Kit (cat. no. MER-004A, Qiagen, Inc., Valencia, CA, USA) according to the manufacturer’s instructions. Detection of inflammatory mediators is based on the standard colorimetric sandwich-based ELISA method using highly specific antibodies. The colour development was recorded with the Labsystems iEMS Reader MF microtiter reader at 450 and 570 nm.

### 4.11. The Alkaline Comet Assay

To investigate the potential genotoxic effect of 13cRA, naringenin, chrysin, and alendronate on DNA molecule, the comet assay was performed according to the previously described protocol [13]. Briefly, a freshly prepared suspensions of blood cells (5 µL) were mixed with 100 µL of 0.5% low melting point (LMP) agarose and spread on microscope slides precoated with 300 µL of 0.6% normal melting point (NMP) agarose. After solidification on ice, the slides were covered with 0.5% LMP agarose, and cells were lysed in a detergent solution (2.5 M NaCl, 100 mM Na2EDTA, 10 mM Tris, 1% sodium sarcosinate, 1% Triton X-100, 10% dimethyl sulfoxide, pH 10) overnight at 4 °C. Following lysis, the slides were placed into an alkaline solution (300 mM NaOH, 1 mM Na2EDTA, pH 13) for 20 min at 4 °C to allow DNA unwinding, and then the electrophoresis is carried out for 20 min at 1 V/cm. Finally, the slides are rinsed with the neutralization buffer (0.4 M Tris buffer (pH 7.5), 5 min, three times), stained with ethidium bromide (20 µg/mL) and covered with a coverslip. The slides were stored in sealed boxes at 4 °C until analysis. For the evaluation of DNA damage, a total of 100 randomly captured comets from each slide were examined at 250× magnification using an epifluorescence microscope (Zeiss, Jena, Germany) equipped with a sensitive black and white camera and connected to an image analysis system (Comet Assay II; Perceptive Instruments Ltd., Suffolk, UK). A computerized image analyser was used to acquire images, compute the integrated intensity profile for each cell, estimate the comet cell components and evaluate the range of derived parameters. To quantify DNA damage, three comet parameters were evaluated: tail length, tail intensity (% of DNA in tail), and tail moment. Tail moment was calculated as (tail length × % of DNA in tail)/100. 

### 4.12. Statistical Analyses 

All data were analysed by a nonparametric Kruskal–Wallis test. Further analysis of the differences between the groups was made with Multiple comparisons of mean ranks for all groups. Statistical analyses were performed using STATISTICA 13 software (StatSoft, Tulsa, OK, USA). The data were considered significant at *p* < 0.05. 

## 5. Conclusions

The implementation of a chrysin- and naringenin-enriched diet could be recommended for patients on RA therapy to help reduce 13cRA-induced OS, oestrogen deficiency, and bone loss. Both flavonoids improved bone quality, bone resorption, and enhanced the rate of bone formation via the antioxidative, anti-inflammatory, and phytoestrogenic activity, without toxic effects that are observed during alendronate administration. This study demonstrated that in a bone microenvironment chrysin and naringenin may successfully counteract OS and inflammation, and consequently reduce the level of tissue damage and the development of chronic diseases such as OP.

## Figures and Tables

**Table 1:** Effects of naringenin, chrysin, or alendronate on femur physical characteristics in rats with retinoic-acid-induced osteoporosis.

Groups ^a^	Femur Physical Characteristics (cm/100 g) (X ± SE)						
	AP ϕ Proximal Epiphysis	ML ϕ Proximal Epiphysis	AP ϕ Mid-Diaphysis	ML ϕ Mid-Diaphysis	AP ϕ Distal Epiphysis	ML Distal Epiphysis	Femur Length Trochanter-Condyle
**Control**	0.153 ± 0.004	0.223 ± 0.010	0.129 ± 0.004	0.172 ± 0.021 ^◊^	0.154 ± 0.009	0.207 ± 0.015	1.405 ± 0.050
**13cRA**	0.137 ± 0.009	0.214 ± 0.011	0.118 ± 0.005	0.141 ± 0.010 *	0.150 ± 0.008	0.213 ± 0.009	1.325 ± 0.044
**Naringenin**	0.152 ± 0.004	0.209 ± 0.009	0.136 ± 0.009	0.148 ± 0.010	0.153 ± 0.010	0.207 ± 0.016	1.489 ± 0.065 ^◊^
**Chrysin**	0.140 ± 0.010	0.198 ± 0.012	0.120 ± 0.009	0.145 ± 0.019	0.144 ± 0.017	0.193 ± 0.011	1.389 ± 0.102
**Alendronate**	0.146 ± 0.004	0.222 ± 0.005	0.128 ± 0.005	0.149 ± 0.004	0.169 ± 0.015	0.228 ± 0.001	1.390 ± 0.036

^a^ Rats were administered *ig* with retinoic acid suspension (80 mg/kg) once daily for 14 days. After inducing osteoporosis with 13cRA, rats were administered *ig* with naringenin (100 mg/kg), chrysin (100 mg/kg), or alendronate (40 mg/kg), once daily for the next 14 days. Results are expressed as means of seven samples per group (*n* = 7) ± SEM. *—Statistically significant when compared with control (* *p* < 0.05); ^◊^—Statistically significant when compared with 13cRA (^◊^ *p* < 0.05).

**Table 2:** Effects of naringenin, chrysin, or alendronate on enzymatic biochemical parameters in blood of rats with retinoic-acid-induced osteoporosis.

Groups ^a^	Biochemical Parameters-Enzymes (X ± SE)					
	AST (U/L)	ALT (U/L)	ALP (U/L)	GGT (U/L)	LDH (U/L)	Amylase (U/L)
**Control**	78.33 ± 7.73	46.40 ± 9.52	170.13 ± 24.54 ^◊^	0.00 ± 0.00	464.50 ± 130.97	1751.00 ± 609.96
**13cRA**	96.16 ± 13.34	43.16 ± 4.66	224.00 ± 15.01 *	0.66 ± 0.51	725.66 ± 157.59 *	2046.16 ± 213.69
**Naringenin**	74.00 ± 5.25 ^◊^	41.00 ± 3.89	147.50 ± 30.09 ^◊^	0.33 ± 0.51	201.50 ± 97.65 ^◊◊◊^	1897.00 ± 311.35
**Chrysin**	83.00 ± 4.00	41.00 ± 4.19	186.33 ± 45.60	0.16 ± 0.40	325.16 ± 121.79 ^◊^	1912.16 ± 510.88
**Alendronate**	87.66 ± 7.50	42.00 ± 2.64	115.00 ± 12.00 ^◊◊^	0.33 ± 0.57	402.00 ± 122.74	2184.00 ± 419.06

^a^ Rats were administered *ig* with retinoic acid suspension (80 mg/kg) once daily for 14 days. After inducing osteoporosis with 13cRA, rats were administered *ig* with naringenin (100 mg/kg), chrysin (100 mg/kg), or alendronate (40 mg/kg), once daily for the next 14 days. Number of rats per group: 7. *—Statistically significant compared with control (* *p* < 0.05); ^◊^—Statistically significant compared with 13cRA (^◊^ *p* < 0.05; ^◊◊^ *p* < 0.01; ^◊◊◊^ *p* < 0.001). Abbreviations: AST—aspartate aminotransferase; ALT—alanine aminotransferase; ALP—alkaline phosphatase; LDH—lactate dehydrogenase; GGT—glutamyl transferase.

**Table 3:** Effects of naringenin, chrysin, or alendronate on differential blood count in rats with retinoic-acid-induced osteoporosis.

Groups ^a^	Leukocytes (×10^9^ L^−1^)	Differential Blood Count (%) (X ± SE)				
		*Lymphocytes*	*Monocytes*	*Neutrophils*	*Basophils*	*Eosinophils*
**Control**	2.70 ± 1.17	79.06 ± 7.99	0.42 ± 0.21	13.46 ± 2.97	0.38 ± 0.21	0.61 ± 0.27
**13cRA**	3.88 ± 1.12	83.96 ± 2.74	0.45 ± 0.16	15.18 ± 2.28	0.55 ± 0.17	0.73 ± 0.36
**Naringenin**	3.85 ± 0.47	86.95 ± 2.02	0.38 ± 0.21	11.45 ± 2.37	0.36 ± 0.23	0.85 ± 0.38
**Chrysin**	4.51 ± 1.33	79.55 ± 6.86	0.36 ± 0.22	15.33 ± 3.29	0.46 ± 0.15	1.05 ± 0.25
**Alendronate**	5.35 ± 1.36 *	70.16 ± 3.29	0.86 ± 0.20 *	28.00 ± 4.33 *	1.23 ± 0.50	0.73 ± 0.41

^a^ Rats were administered *ig* with retinoic acid suspension (80 mg/kg) once daily for 14 days. After inducing osteoporosis with 13cRA, rats were administered *ig* with naringenin (100 mg/kg), chrysin (100 mg/kg), or alendronate (40 mg/kg), once daily for the next 14 days. Number of rats per group: 7. *—Statistically significant compared with control (* *p* < 0.05).

**Table 4:** Effects of naringenin, chrysin, or alendronate on oxidative stress markers in liver, kidney, spleen, and ovary tissues in rats with retinoic-acid-induced osteoporosis.

Groups ^a^	MDA (nmol/mg Proteins)			
	Liver	Spleen	Kidney	Ovary
**Control**	10.22 ± 0.56 ^◊^	16.24 ± 1.05	4.09 ± 0.60 ^◊◊^	12.12 ± 1.63 ^◊◊^
**13cRA**	15.58 ± 0.63 *	17.61 ± 1.56	12.33 ± 0.72 **	35.78 ± 3.45 **
**Naringenin**	13.36 ± 0.33	13.66 ± 0.92 ^♦^	3.90 ± 0.46 ^◊◊^	20.06 ± 2.01 ^◊^
**Chrysin**	14.04 ± 0.42	19.11 ± 1.70	4.81 ± 0.53 ^◊◊^	28.99 ± 1.33 *^◊^
**Alendronate**	18.05 ± 0.83 **	23.62 ± 2.15	9.84 ± 0.87 *	37.41 ± 2.14 **
	**GSH (µg/mg proteins)**			
**Control**	8.90 ± 0.71	14.78 ± 2.25	8.46 ± 0.11 ^◊^	0.95 ± 0.15 ^◊^
**13cRA**	5.65 ± 0.54	8.39 ± 0.38	3.61 ± 0.32 *	0.20 ± 0.02 *
**Naringenin**	7.08 ± 1.05	15.11 ± 3.18	10.07 ± 0.54 ^◊^	0.56 ± 0.10
**Chrysin**	7.64 ± 1.63	13.38 ± 1.77	6.75 ± 0.69	1.01 ± 0.09 ^◊^
**Alendronate**	5.09 ± 1.20	12.69 ± 1.27	10.63 ± 0.61 ^◊◊^	0.37 ± 0.20
	**SOD (U/mg proteins)**			
**Control**	3.67 ± 0.69 ^◊^	5.38 ± 0.52	4.07 ± 0.48	15.46 ± 1.81 ^♦♦^
**13cRA**	1.66 ± 0.13 *	4.22 ± 1.14	3.94 ± 0.52	7.44 ± 0.69
**Naringenin**	4.61 ± 0.35 ^◊◊^^♦^	9.03 ± 1.80 ^◊^	4.56 ± 0.86	9.28 ± 1.01 ^♦^
**Chrysin**	2.50 ± 0.22	4.93 ± 1.06	4.06 ± 1.40	11.54 ± 1.81 ^♦^
**Alendronate**	1.77 ± 0.38	5.29 ± 1.37	7.74 ± 1.75	2.46 ± 0.43 **
	**CAT (U/mg proteins)**			
**Control**	4.98 ± 0.42	4.22 ± 0.28	21.99 ± 1.58	14.03 ± 0.06
**13cRA**	4.67 ± 0.61	2.76 ± 0.40	8.94 ± 0.73	11.64 ± 1.73
**Naringenin**	4.45 ± 0.62	4.09 ± 0.87	43.77 ± 5.71 ^◊◊^	15.92 ± 1.85
**Chrysin**	4.70 ± 0.34	2.82 ± 0.33 ^♦^	41.24 ± 4.87 ^◊◊^	24.53 ± 0.24 **^◊^
**Alendronate**	4.08 ± 0.86	6.99 ± 0.56 ^◊^	66.45 ± 4.21 ^◊◊◊^	20.92 ± 1.10 *^◊^

^a^ Rats were administered *ig* with retinoic acid (13cRA) suspension (80 mg/kg) once daily for 14 days. After inducing osteoporosis with 13cRA, rats were administered *ig* with naringenin (100 mg/kg), chrysin (100 mg/kg), or alendronate (40 mg/kg), once daily for the next 14 days. Number of rats per group: 7. Data were analysed by Kruskal–Wallis ANOVA test. *—Statistically significant compared with control (* *p* < 0.05; ** *p* < 0.01). ^◊^—Statistically significant compared with 13cRA (^◊^ *p* < 0.05; ^◊◊^ *p* < 0.01; ^◊◊◊^ *p* < 0.001). ^♦^—Statistically significant compared with alendronate (^♦^ *p* < 0.05; ^♦♦^ *p* < 0.01).

**Table 5:** Parameters analysed and methods used for their determination in osteoporotic rats.

	Parameter	Method
Serum biochemical parameters	Aspartate aminotransferase (AST), Alanine aminotransferase (ALT), Alkaline phosphatase (ALP), Glutamyl transferase (GGT), Amylase Urea, creatinine, Blood glucose levels (glucose), Lactate dehydrogenase (LDH), Total protein and serum Ca and P levels	Colorimetric method
Hematological parameters	Erythrocytes (E), The average cellular volume of erythrocytes (MCV), Haemoglobin (Hgb), Haematocrit (Hct), Mean cell haemoglobin (MCH), Mean cell Haemoglobin concentration (MCHC), Total leukocyte count (L), and The total number of platelets (Plt)	Colorimetric method
Serum Inflammatory cytokines	IL1α, IL1β, IL2, IL4, IL6, IL10, IL12, IL17A, IFNγ, TNFα, GM-CSF, and RANTES	ELISA method
Biochemical bone parameters	Ca and P analyses	Atomic absorption spectrophotometry
	Bone mineral density (BMD)	Dual-energy X-ray absorptiometry (DXA).
	Serum markers of bone turnover Osteocalcin (OC) β-CrossLaps	Electrochemiluminescence “ECLIA” method
Bone physical parameters	Relative bone weight index, Length of the bone (large trochanter-condyle), The distal and proximal epiphyseal diameters of the femur were measured in mediolateral (ML) and anteroposterior directions (AP)	Digital scale Small bone caliper
Histological parameters	Histological analyses	Haematoxylin and eosin (HE) methods
Oxidative stress parameters	Lipid peroxidation level (MDA), Glutathione level (GSH) Superoxide dismutase (SOD) activity Catalase (CAT) activity	Colorimetric method
	DNA damage	Alkaline Comet Assay

## Data Availability

The original contributions generated for this study are included in the article; further inquiries can be directed to the corresponding author.
